# A novel easy-to-use index to predict institutionalization and death in older population – a 10-year population-based follow-up study

**DOI:** 10.1186/s12877-023-03760-1

**Published:** 2023-02-07

**Authors:** Elisa Heikkilä, Marika Salminen, Anna Viljanen, Taina Katajamäki, Marja-Kaisa Koivula, Kari Pulkki, Raimo Isoaho, Sirkka-Liisa Kivelä, Matti Viitanen, Minna Löppönen, Tero Vahlberg, Mikko S. Venäläinen, Laura L. Elo, Laura Viikari, Kerttu Irjala

**Affiliations:** 1 Faculty of Medicine, Department of Clinical Medicine, Unit of Clinical Chemistry, Turku University, FI-20014 University of Turku, 20521 Turku, Finland; 2 Tykslab, Laboratory Division, Turku University Hospital, Hospital District of Southwest Finland, Turku, Finland; 3 Faculty of Medicine, Department of Clinical Medicine, Unit of Family Medicine, University of Turku and Turku University Hospital, 20014 Turku, Finland; 4 City of Turku, Welfare Division/Turku City Hospital, Kunnallissairaalantie 20, 20700 Turku, Finland; 5 Municipality of Lieto, Health Care Center, 21420 Lieto, Finland; 6 Faculty of Medicine, Department of Geriatrics, Turku City Hospital, University of Turku, Kunnallissairaalantie 20, 20700 Turku, Finland; 7 HUS Diagnostic Center, Helsinki University Hospital, Hospital District of Helsinki and Uusimaa (HUS), Helsinki, Finland; 8 Diagnostic Center, Clinical Chemistry and Hematology, Helsinki University Hospital and University of Helsinki, Helsinki, Finland; 9 City of Vaasa, Social and Health Care, 65101 Vaasa, Finland; 10 Faculty of Pharmacy, Division of Social Pharmacy, University of Helsinki, 00014 Helsinki, Finland; 11 City of Raisio, Social and Health Care for Elderly, 21200 Raisio, Finland; 12 Faculty of Medicine, Department of Clinical Medicine, Unit of Biostatistics, University of Turku, Turku, Finland; 13 Turku Bioscience Centre, University of Turku and Åbo Akademi University, 20520 Turku, Finland; 14 Institute of Biomedicine, University of Turku, Turku, Finland

**Keywords:** Institutionalization, Mortality, Aged, Index

## Abstract

**Background:**

Various indexes have been developed to estimate the risk for mortality, institutionalization, and other adverse outcomes for older people. Most indexes are based on a large number of clinical or laboratory parameters. An index based on only a few parameters would be more practical to use in every-day clinical practice. Our aim was to create an index to predict the risk for mortality and institutionalization with as few parameters as possible without compromising their predictive ability.

**Methods:**

A prospective study with a 10-year follow-up period. Thirty-six clinical and fourteen laboratory parameters were combined to form an index. Cox regression model was used to analyze the association of the index with institutionalization and mortality. A backward statistical method was used to reduce the number of parameters to form an easy-to-use index for predicting institutionalization and mortality.

**Results:**

The mean age of the participants (*n* = 1172) was 73.1 (SD 6.6, range 64‒97) years. Altogether, 149 (14%) subjects were institutionalized, and 413 (35%) subjects deceased during the follow-up. Institutionalization and mortality rates increased as index scores increased both for the large 50-parameter combined index and for the reduced indexes. After a backward variable selection in the Cox regression model, three clinical parameters remained in the index to predict institutionalization and six clinical and three laboratory parameters in the index to predict mortality. The reduced indexes showed a slightly better predictive value for both institutionalization and mortality compared to the full index.

**Conclusions:**

A large index with fifty parameters included many unimportant parameters that did not increase its predictive value, and therefore could be replaced with a reduced index with only a few carefully chosen parameters, that were individually associated with institutionalization or death.

**Supplementary Information:**

The online version contains supplementary material available at 10.1186/s12877-023-03760-1.

## Background

Various indexes have been developed to estimate the risk for adverse outcomes such as mortality, institutionalization, worsening health status, hospitalization, increased falls, morbidity, and dependence in older people. Different indexes have been formed using clinical frailty scales [1–3], laboratory data [4–9] or a combination of these [6–9]. We have earlier demonstrated that an index based on clinical deficits [10] as well as an index based on routine laboratory parameters [11] can both be used in predicting mortality in Finnish elderly population in 10- and 18-year follow-ups. However, the index based solely on laboratory tests did not predict institutionalization [11]. The clinical index predicted both mortality and institutionalization [10].

The clinical parameters give information on the current health status of a person and the person’s functional abilities, and abnormal results in blood tests may reflect the subclinical health deficits or diseases before they become clinically manifested. These preclinical conditions may contribute to the risk of mortality and other adverse health outcomes. [5, 7–9, 12–14]. A combination of clinical and laboratory parameters might be an optimal solution to assess especially older people’s risk of mortality.

Other studies have shown that indexes formed by combining at least 30 deficits are strongly associated with the risk of death, institutionalization, and worsening health status, although different indexes include different variables [1, 10, 15, 16]. Our previous study compared different clinical frailty indexes on the same population, and found simple and fast frailty indexes to be comparable with a multidimensional and time-consuming frailty index in predicting mortality among community-dwelling Finnish older people [16]. Also, simple self-reported measures of walking ability and self-rated health seemed to be comparable with frailty indexes in predicting institutionalization among community-dwelling older people in a 10-year follow-up [10]. These results suggest that it might be possible to form an index that only includes a few parameters, and still has a good predictive ability on mortality and institutionalization. Especially in primary health care, an index with a smaller number of parameters would be more practical to use. In large indexes, some parameters, such as the different activities of daily living, may correlate significantly with each other, and thus, their number could possibly be reduced without compromising the predictive ability of the index. The aim of this study was to first form a combined clinical and laboratory index, and then to form an easy-to-use index with reduced number of parameters for elderly patient care that would be faster and easier to obtain.

## Methods

### Study design and population

This study is part of a longitudinal epidemiological study carried out in the municipality of Lieto in Southwestern Finland [17]. All persons born in or prior to the year 1933 (*n* = 1596) were invited to participate in the baseline examination which was carried out between March 1998 and September 1999. Of those eligible, 63 died before they were examined, and 273 refused or did not respond, leaving 1260 (82%) participants, 533 men and 727 women. They were followed-up for institutionalization and mortality for 10 years. Participants no longer living in Lieto at the end of the follow-up period (*n* = 86) were excluded from the analyses predicting institutionalization, as it was not possible to ascertain whether they were institutionalized in another municipality or continued living at home. Sixty-eight participants already living in institutional care at the start of the study were excluded from the institutionalization analyses. Also, participants with missing data of more than five percent of parameters included in the indexes were excluded leaving 1054 and 1172 participants for the analyses predicting institutionalization and mortality, respectively.

### Measurements

At baseline in 1998 to 1999, venous blood samples were obtained with minimal stasis between 8 and 10 am after overnight fast at Lieto health center. Fresh samples were analyzed at the Central Laboratory of Turku University Hospital. All participants were given verbal and written instructions before laboratory visit. Data for the clinical parameters were gathered from a doctor’s clinical examination including a comprehensive interview and a survey of patient records at the baseline [10].

### Mortality

Data from all participants who died by the end of 2008 were obtained from the official Finnish Cause of Death Registry using unique personal identification numbers.

### Institutionalization

Institutionalization was defined as permanent entry into a nursing home of which the data were gathered from the municipality’s electronic patient record system and coded by month and year of entry.

### The combined clinical and laboratory index

We combined the 36 clinical and 14 laboratory parameters, that have been used in our earlier studies separately, to form an index with possibly a better predicting ability on both mortality and institutionalization. The clinical parameters included 36 symptoms, signs, and disabilities that are used in Rockwood’s frailty index [15, 18] and can be seen in Appendix 1. The laboratory parameters that we chose are carefully selected so that they do not overlap significantly as risk indicators but reflect the health status of different organ systems and that they are routinely tested when examining elderly patients in Finland. The laboratory analytes and their reference ranges or cut-off values are shown in Appendix 2 and in our previous article on a laboratory index [11].

The combined index (CI) was constructed by coding each deficit or laboratory analyte as either 0 or 1; 1 indicates that the person had a certain deficit, or a laboratory value was above or below the normal range or cut-off. The sum of these values was then divided by the total number of the deficits and analytes resulting in a score ranging from 0 to 1 for each individual. To compare the adverse outcomes of individuals with different CI scores, we divided the participants in three categories using established cut-points that have been used in frailty indexes [3] (1. CI ≤ 0.085, 2. CI 0.085–0.2499, 3. CI ≥ 0.25).

### Statistical analyses

Hazard ratios (HRs) and their 95% confidence intervals for all-cause mortality and institutionalization were calculated using Cox proportional hazard models. The follow-up periods were calculated from the baseline measurements to the end of the follow-up period of 10 years or to the death of the individual. Death was used as a competitive factor in the analyses for institutionalization. Both unadjusted and age- and gender-adjusted analyses were conducted. The forming of the reduced indexes was started by selecting those clinical and laboratory parameters that were associated with institutionalization or mortality in the univariable Cox regression analysis. A backward stepwise Cox regression analysis (exclusion criteria, *p* ≥ 0.05) was performed to identify the parameters which best predicted mortality and institutionalization and to reduce the number of parameters in the index [19]. Reduced indexes were also calculated by including age (64–69, 70–74, 75–79, 80 or over) and gender to the index. Clinical and laboratory parameters were scored with 0 or 1 points, female gender was scored with 1 point for the institutionalization index and male gender with 1 point for the mortality index, and age was scored with 0 point for ages 64–69, 1 point for ages 70 to 74, 2 points for ages 75 to 79 and 3 points for the age of 80 or more based on the parameter estimates of the stepwise Cox regression model and statistical testing in the effect of different weights on the index. This resulted a range of 0 to 7 for the combined index to predict institutionalization and a range of 0 to 13 to predict mortality. Receiver-operating characteristic (ROC) curves were used to define the cut-off points for reduced indexes to predict institutionalization and mortality [20]. The optimal cut-off points were chosen using Youden index [21]. Sensitivity, specificity, positive predictive value and negative predictive value for cut-off points were calculated. Kaplan–Meier survival curves for institutionalization and mortality during the 10-year follow-up were done to compare participants below and above the cut-off points [19]. *P*-values less than 0.05 were considered statistically significant. All statistical analyzes were performed using SAS System for Windows, version 9.4 (SAS Institute Inc., Cary, NC, USA).

## Results

### Baseline characteristics

The mean age of the participants was 73.1 years (range 64 to 97 years). The majority, 57 percent of the participants were female. More detailed baseline characteristics of 1172 study participants are shown in Appendix 3.

### The combined index to predict institutionalization

Altogether 149 (14%) subjects were institutionalized during the 10-year follow-up.

Higher index score of the 50-parameter combined index was associated with an increased risk of institutionalization in the 10-year follow-up. Both groups with CI 0.085–0.2499 and CI 0.25 or over had statistically significantly higher risk of institutionalization compared to the group with CI less than 0.085. These associations persisted after adjustments for age and gender (Table 1).

**Table 1:** Hazard ratios (HR) and their 95% confidence intervals (CI) (in parentheses) of the combined clinical and laboratory index for institutionalization (*n* = 1054) and mortality (*n* = 1172) during the 10-year follow-up

	Institutionalization					Mortality				
Index score	n	Unadjusted HR (95% CI)	*P*-value	Adjusted* HR (95% CI)	*P*-value	n	Unadjusted HR (95% CI)	*P*-value	Adjusted* HR (95% CI)	*P*-value
< 0.085	207	1		1		225	1		1	
0.085–0.2499	653	2.42 (1.30–4.52)	0.006	1.88 (1.00–3.50)	0.049	711	2.23 (1.54–3.23)	< 0.001	2.08 (1.43–3.02)	< 0.001
≥ 0.25	194	6.64 (3.49–12.61)	< 0.001	2.95 (1.46–5.96)	0.003	236	9.39 (6.43–13.71)	< 0.001	5.64 (3.79–8.40)	< 0.001

^*^Values are adjusted for age and gender

### The combined index to predict mortality

Altogether 413 (35%) subjects deceased during the 10-year follow-up.

Higher index score of the 50-parameter combined index was associated with an increased risk of death in the 10-year follow-up. Both groups with CI 0.085–0.2499 and CI 0.25 or over had a statistically significantly higher risk of death compared to the group with CI less than 0.085. These associations remained statistically significant when adjusted for age and gender (Table 1).

### The reduced easy-to-use index to predict institutionalization

**Fig. 1:**
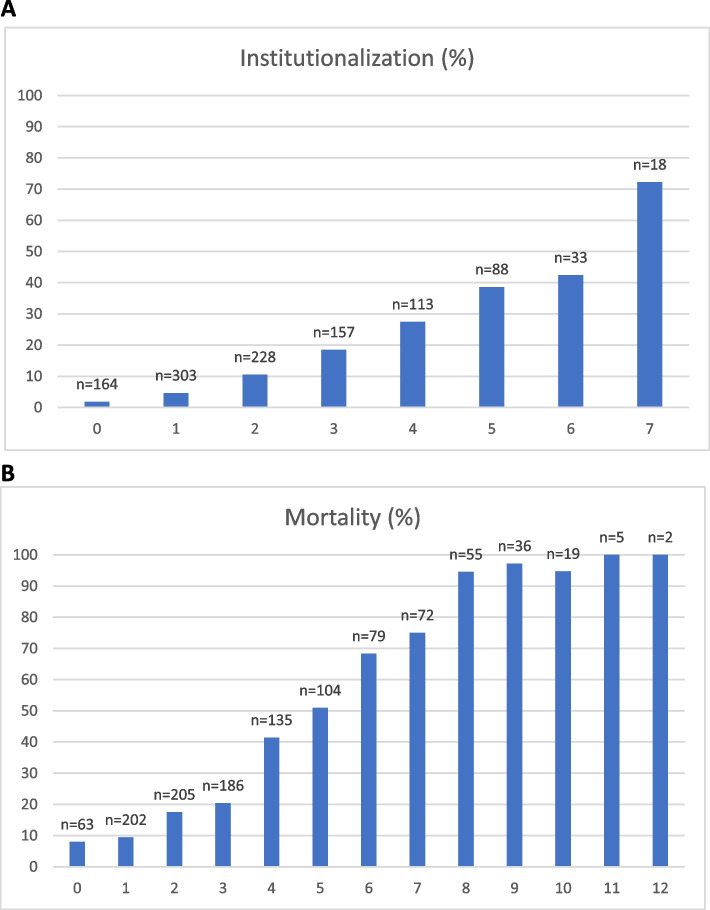
Institutionalization (**A**) and mortality (**B**) (rates) by each index score during the 10-year follow-up

**Fig. 2:**
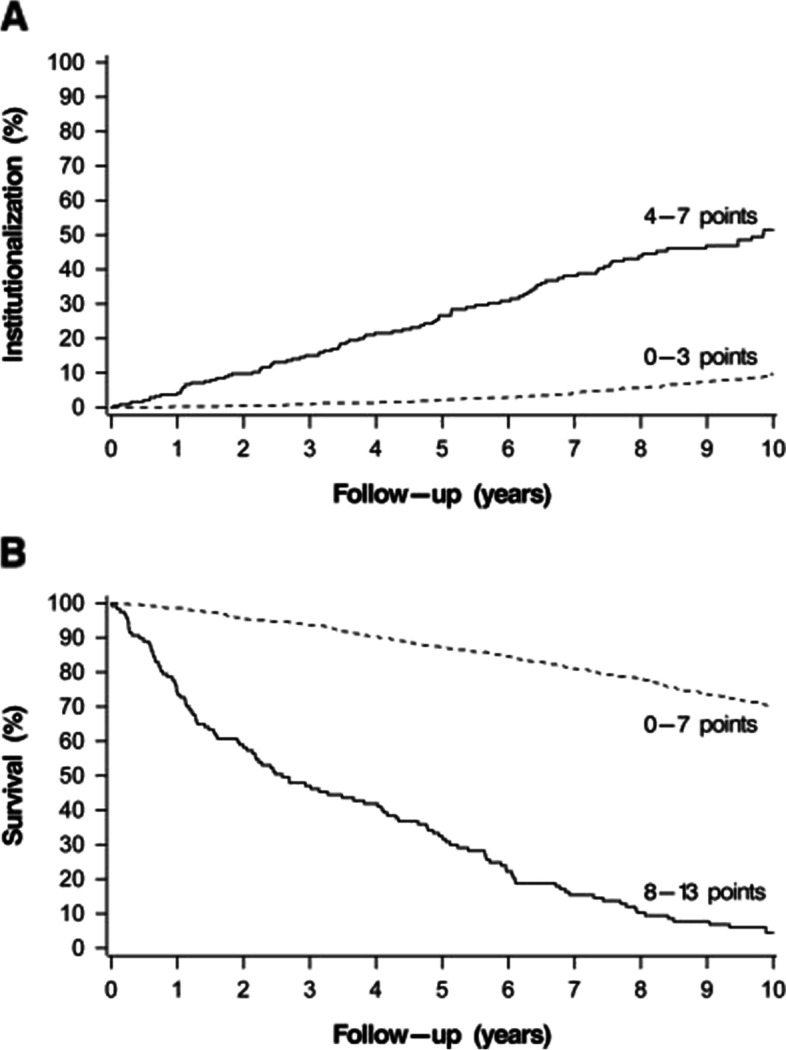
Rates of institutionalization by the reduced index with the cut-off score ≥ 4 (**A**) and mortality by the reduced index with the cut-off score ≥ 8 (**B**) during the 10-year follow-up. Age and gender are included in both reduced indexes

Twenty-five clinical parameters from the large, combined index were independently associated with institutionalization in the univariable analysis. After a backward variable selection in the Cox regression model, the parameters that were left for the reduced index to predict institutionalization were the need for help with preparing meals, the need for help with heavy household chores and the need for help with moving about inside house. Female gender and increasing age were significant predicting factors for institutionalization and adding these factors to the index improved its predictive ability (Table 2). Each factor was coded either 0 or 1; 0 indicates that the person did not need help with the task and 1 that the person needed help. One point was given for female gender and 0 to 3 points for increasing age resulting in a range of 0 to 7 for the index score. The parameters and their scoring can be seen on Table 3. The best cut-off limit for the increased risk of institutionalization was found to be ≥ 4 points. The higher the index score, the higher percentage of our study population were institutionalized during the 10-year-follow-up period. This can be seen on Fig. 1a. Figure 2a shows Kaplan–Meier survival curve by the cut-off limit of 4 points.

**Table 2:** AUC-values, cut-off limits and their sensitivities, specificities and positive and negative predictive values for the 50-parameter combined index and for the reduced indexes in predicting 10-year institutionalization and mortality

	**AUC (95% CI)**	**Cut-off limit (scale)**	**Sensitivity (%)**	**Specificity (%)**	**PPV (%)**	**NPV (%)**
**Institutionalization**						
50-parameter combined index	0.71 (0.66–0.75)	≥ 0.25* (0–1)	38.3	84.9	29.4	89.3
Reduced index without age and gender	0.74 (0.70–0.78)	≥ 2** (0–3)	71.0	71.8	30.2	93.5
Reduced index with age and gender	0.78 (0.75–0.82)	≥ 4** (0–7)	74.7	69.4	29.6	94.1
**Mortality**						
50-parameter combined index	0.77 (0.74–0.80)	≥ 0.25* (0–1)	42.5	92.0	74.2	74.7
Reduced index without age and gender	0.79 (0.77–0.82)	≥ 5** (0–9)	61.8	84.2	69.5	79.2
Reduced index with age and gender	0.83 (0.80–0.85)	≥ 8** (0–13)	77.0	75.8	64.9	85.1

Abbreviations: *AUC* Area under the curve, *CI* Confidence interval, *PPV* Positive predictive value, *NPV* Negative predictive value

^*^Habitual cut-off limit for a frailty index

^**^Best cut-off limit defined by Youden-index

**Table 3:** The parameters and their scoring for the reduced indexes

**Institutionalization**	**Index points**
Needs help with preparing meals	1
Needs help with heavy household chores	1
Needs help with moving about inside house	1
Female gender	1
Age 70 to 74	1
Age 75 to 79	2
Age 80 or more	3
**Total index score**	**0–7**
**Mortality**	**Index points**
Elevated or decreased blood hemoglobin value	1
Elevated plasma c-reactive protein level	1
Elevated or decreased plasma sodium level	1
Needs help with preparing meals	1
Needs help with heavy household chores	1
Difficulties carrying or lifting light loads	1
Limited kind of amount of activity	1
Diabetes mellitus	1
Heart disease	1
Male gender	1
Age 70 to 74	1
Age 75 to 79	2
Age 80 or more	3
**Total index score**	**0–13**

### The reduced easy-to-use index to predict mortality

Thirty-seven parameters from the large, combined index were independently associated with mortality. The reduced index for mortality included three laboratory analytes and six clinical parameters. The parameters were elevated or decreased blood hemoglobin value, elevated plasma c-reactive protein level, and elevated or decreased plasma sodium level, the need for help with preparing meals, the need for help with heavy household chores, difficulties carrying or lifting light loads, limited kind of amount of activity, diabetes mellitus and heart disease. One point was given for each of these deficits or a laboratory value outside the reference ranges. Increasing age and male gender were significant predicting factors for mortality so, similarly to the index predicting institutionalization, 0 to 3 points was given for age and 1 point for male gender. This resulted in a score ranging from 0 to 13 with the best cut-off limit being ≥ 8 points for the prediction of a person being at an increased risk of death. The parameters and their scoring can be seen on Table 3. Increasing index score increased deaths in our study population during the 10-year-follow-up period as seen on Fig. 1b. Figure 2b shows Kaplan–Meier survival curve by the cut-off limit of 8 points.

## Discussion

With the increasing elderly population, finding the individuals most at risk of institutionalization or death could help in targeting heath care interventions and increase older people’s survival time at home. We used a statistical method to find a combination of a few parameters that best predicted mortality and institutionalization out of large number of variables. This way we could create indexes that take little time to implement, and still have a good predictive ability.

Both clinical and laboratory data were used in the formation of the combined and reduced indexes. The clinical data included deficits that reflect several aspects of the person´s health status such as mobility, daily functions and diagnosed diseases. For the laboratory parameters we wanted to choose only those that are routinely tested also in primary health care so that the index could be easily introduced in clinical practice.

The combined clinical and laboratory index was not better for predicting institutionalization compared to the clinical frailty index alone that was studied in the previous study on the same population [10]. No laboratory parameters were left in the index predicting institutionalization when reducing the parameters by statistical methods, which is consistent with the results of our earlier study that a laboratory-based index could not predict institutionalization [11]. Routine laboratory tests cannot predict dementia and cognitive impairment which are the most common causes of institutionalization [22–27].

The three parameters that remained in the reduced index for institutionalization were need for help with preparing meals, need for help with heavy household chores, and need for help with moving about inside the house. Being able to prepare meals independently is one of the instrumental activities of daily living (IADL), that reflect the person’s ability to carry out daily activities, and their cognitive function. Earlier studies have shown dependence in daily activities to be a predictor of morbidity and mortality in elderly populations [28]. Need for help with moving about inside house is also consistent with the earlier finding that self-reported walking ability predicts a person´s risk for institutionalization [10].

The combined clinical and laboratory index was better at predicting mortality than the clinical or laboratory index alone. The clinical parameters left in the index as predicting factors for mortality were need for help with preparing meals, limited kind of amount of activity, need for help with heavy household chores and difficulties carrying or lifting light loads. Two of them reflect the physical capacity of the person. Many studies have found physical activity to have a positive effect on health and reduce mortality [29, 30]. Limited kind of amount of activity was a predicting factor for mortality and refers also to other than physical activity, including interests and hobbies. In addition to these, diabetes and heart disease were left as predicting factors in the index predicting mortality.

The laboratory parameters that were found significant in predicting mortality have all been shown to predict mortality also independently in other studies [28–34]. Anaemia increases mortality in the older population, and the lowest mortality has been found at normal haemoglobin levels [31, 32]. Elevated c-reactive protein level has been shown to predict increased risk of all-cause and cardiovascular mortality in the general population [33]. Studies have found associations with sodium levels and mortality in general population [34, 35], hospitalized patients [36], and in patients with chronic kidney disease [37].

The strengths of our study are the large sample size, the good participation rate of 82% and a long follow-up period that enable broad generalizability of the results. The data comes from a community-based representative sample of the Finnish population. The gender distribution of the participants is comparable to the distribution of this age group in the whole country [38], and the prevalence of cognitive impairment is similar to the estimated prevalence in the whole country [39].

Persons still living at home after the 10-year follow-up were considered not institutionalized in our analyses which can be considered a limitation of our study since some of them will be institutionalized during their lifetime.

Our results showed that a large index with fifty parameters included many unnecessary parameters that did not increase its predictive value, and therefore could be replaced with a reduced index with only a few carefully chosen parameters, that were individually associated with institutionalization or death. The reduced indexes had even a slightly better predictive ability in comparison to the 50-parameter combined indexes. These indexes with only a few clinical questions in addition to three basic laboratory tests would take very little time in a doctor´s appointment and thus could be used to screen older people. This kind of short intervention could be done during any health care contact of an older person. The indexes could also potentially be calculated automatically if the necessary information was collected in the electronic patient records similarly to electronic frailty indexes that are automatically populated from routine collected data contained within the electronic patient records [40].

Further validation of the indexes in another population is needed.

## Conclusions

A large index with fifty parameters included many unimportant parameters that did not increase its predictive value for institutionalization or mortality, and therefore could be replaced with a reduced index with only a few carefully chosen parameters, that were individually associated with institutionalization or death. An index including only three clinical parameters could predict institutionalization, and an index including three laboratory analytes and six clinical parameters could predict mortality. Their good predictive ability in addition to the small number of parameters could make them easily applicable instruments in clinical settings.

## Supplementary Information

**Additional file 1:** **Appendix 1.** Clinical parameters used for the indexes. **Appendix 2.** Laboratory analytes and their reference ranges used for the indexes. **Appendix 3.** Baseline characteristics of study participants (n=1172)

## Data Availability

The datasets used and/or analysed during the current study are available from the corresponding author on reasonable request.

## Abbreviation

CI: Combined index
