# Supplementary material for: A novel easy-to-use index to predict institutionalization and death in older population – a 10-year population-based follow-up study
Source: BMC Geriatr. 2023 Feb 7;23:80. doi: 10.1186/s12877-023-03760-1 (PMC9903495; doi:10.1186/s12877-023-03760-1)
Supplement: Supplementary file 1 — Additional file 1: Appendix 1. Clinical parameters used for the indexes. Appendix 2. Laboratory analytes and their reference ranges used for the indexes. Appendix 3. Baseline characteristics of study participants (n=1172) [file 12877_2023_3760_MOESM1_ESM.docx]

**Appendix 1** Clinical parameters used for the indexes

| Needs help with toileting | Shortness of breath | |  |
| --- | --- | --- | --- |
| Needs help with dressing and undressing | Angina pectoris | |  |
| Needs help with preparing meals | Other medical problems^b^ | |  |
| Needs help with house work | No regular physical exercise | |  |
| Needs help with heavy household chores | Vision problem | |  |
| Needs help with personal care | Hearing problem | |  |
| Needs help with moving about inside house | Feeling hopeless | |  |
| Arthritis or rheumatism | Emotional problem | |  |
| High blood pressure | Memory problem | |  |
| Chronic bronchitis or emphysema | Bodily pain | |  |
| Diabetes mellitus | Speech problem | |  |
| Heart disease^a^ | Resting tremor | |  |
| Cancer | Five or more medications | |  |
| Stomach or intestinal ulcers | Difficulties carrying or lifting light loads | |  |
| Suffers from the effect of stroke | Mobility problem | |  |
| Urinary incontinence | Limited kind of amount of activity | |  |
| Stool incontinence | | Feeling tired all the time | |
| Hip or femoral fracture | | Weight loss | |

^a^Known heart disease at baseline (ICD-10: I20-I25, I48, I49)

^b^Other disease at baseline (ICD-10: E03, E05, G20, G35, J44-J46, M15-M17, M47)

**Appendix 2** Laboratory analytes and their reference ranges used for the indexes

|  | Men | Women |  |
| --- | --- | --- | --- |
| Hemoglobin (g/L) | 128-168 | 117-153 |  |
| Albumin (g/L) | 36.1-47.5 | 34.8-46.1 |  |
| Calcium (mmol/L) | 2.17-2.47 | 2.17-2.47 |  |
| Urate (µmol/L) | 180-420 | 130-340 |  |
| TSH (mU/L) | 0.4-4.5 | 0.4-4.5 |  |
| Creatinine (µmol/L) | < 135 | < 125 |  |
| Ferritin (µg/L) | 20-240 | 10-100 |  |
| CRP (mg/L) | < 3 | < 3 |  |
| Sodium (mmol/L) | 136-144 | 136-144 |  |
| Potassium (mmol/L) | 3.5-4.8 | 3.5-4.8 |  |
| Glucose (mmol/L) | 4.0-6.4 | 4.0-6.4 |  |
| ALT (U/L) | < 50 | < 35 |  |
| ALP (U/L) | < 300 | < 300 |  |
| LDL cholesterol (mmol/L) | < 3.5 | < 3.5 |  |

Abbreviations: TSH, thyroid stimulating hormone; CRP, C-reactive protein; ALT, alanine aminotransferase; ALP, alkaline phosphatase; LDL, low-density lipoprotein.

**Appendix 3** Baseline characteristics of study participants (n=1172)

|  | *n* (%) |
| --- | --- |
| Age, mean (SD), range | 73.1 (6.6), 64-97 |
| Age |  |
| 64-74 | 753 (64) |
| 75-84 | 335 (29) |
| ≥85 | 84 (7) |
| Female | 669 (57) |
| Living alone | 345 (29) |
| Education |  |
| Basic^a^ or less than basic | 1039 (89) |
| More than basic | 133 (11) |
| MMSE ≤26 | 305 (26) |
| Body mass index, kg/m^2^ |  |
| <20 | 51 (4) |
| 20-24.9 | 314 (27) |
| 25-29.9 | 519 (44) |
| 30-34.9 | 221 (19) |
| ≥35 | 64 (5) |

^a^Six years of elementary school

MMSE, Mini-Mental State Examination
